# Transarterial chemoembolization (TACE) using mitomycin and lipiodol with or without degradable starch microspheres for hepatocellular carcinoma: comparative study

**DOI:** 10.1186/s12885-018-4099-x

**Published:** 2018-02-14

**Authors:** Tatjana Gruber-Rouh, Cornelia Schmitt, Nagy N. N. Naguib, N. A. Nour-Eldin, Katrin Eichler, Martin Beeres, Thomas J. Vogl

**Affiliations:** Institute for Diagnostic and Interventional Radiology, Johann Wolfgang Goethe-University Frankfurt, Theodor-Stern-Kai 7, 60590 Frankfurt am Main, Germany

**Keywords:** Chemoembolization, HCC, Lipidol, DSM, Survival data, Local control

## Abstract

**Background:**

To evaluate survival data and local tumor control after transarterial chemoembolization in two groups with different embolization protocols for the treatment of HCC patients.

**Methods:**

Ninty-nine patients (mean age: 63.6 years), 78 male (78.8%) with HCC were repeatedly treated with chemoembolization in 4-week-intervals. Eighty-eight patients had BCLC-Stage-B and in 11 patients, chemoembolization was performed for bridging (BCLC-Stage-A). In total, 667 chemoembolization treatments were performed (mean 6.7 treatments/patient). The administered chemotherapeutic agent included mitomycin. For embolization, lipiodol only (*n* = 51;51.5%; mean age 63.8 years; 38 male), or lipiodol plus degradable starch microspheres (DSM) (*n* = 48; 48.5%; mean age 63.4 years; 40 male) were used. The local tumor response was assessed by MRI using Response Evaluation Criteria in Solid Tumors 1.1 (RECIST 1.1). Patient survival times were evaluated using Kaplan-Meier curves and log-rank tests.

**Results:**

The local tumor control in the lipiodol-group was: PR (partial response) in 11 (21.6%), SD (stable disease) in 32 (62.7%) and PD (progressive disease) in 8 cases (15.7%). In the lipiodol-DSM-group, PR was seen in 14 (29.2%), SD in 22 (45.8%), and PD in 12 (25.0%) individuals (*p* = 0.211). The median survival of patients after chemoembolization with lipiodol was 25 months and in the lipiodol-DSM-group 28 months (*p* = 0.845).

**Conclusion:**

Our data suggest a slight benefit of the use of lipiodol and DSM in comparison of using lipiodol only for chemoembolization of HCC in terms of local tumor control and survival data, this trend did not reach the level of significance.

## Background

Hepatocellular carcinoma (HCC) is the most common primary malignancy of the liver [1]. Surgical resection, liver transplantation, and tumor ablation, e.g. using radio frequency or thermic techniques, are the only curative options for patients with HCC [2]. However, all options have limited applicability: only few patients (25%) are appropriate candidates for transplantation and there is a lack of liver donors. While surgical resection is the first line therapy for primary liver cancer, only 10-30% of the patients are eligible for surgery with curative intent. This can be explained by frequent presence of extensive disease and poor liver function due to cirrhosis [2–4]. Furthermore, local tumor ablation using microwave or thermal treatment with radio frequency and kryotherapy respectively, is predominantly effective for tumors with a diameter less than 5 cm [5].

Patients suffering from HCC greater 5 cm diameter, embolization and chemotherapy are established treatment options [6, 7]. Treatment with embolization and chemotherapy can be conducted individually by using transarterial embolization (TAE), transarterial chemoembolization (TACE), or TACE in combination with drug-eluting-beats (DEB-TACE). TACE is a treatment option for non-resectable HCC [6, 7], resulting in a high cytotoxic effect after usage of chemotherapeutic drugs and causing ischaemia due to the use of embolization particles [2, 4, 8]. Consequently, TACE might be a useful treatment option to improve outcomes of potentially curative therapies or as a bridging therapy to liver transplantation [8].

Although TACE in HCC patients has already been intensively investigated, literature is still inconclusive about the relative effectiveness of different embolization agents [9].

The aim of this retrospective study was to compare tumor response and survival time after transarterial chemoembolization in HCC patients by using two different chemoembolization agent protocols (lipiodol-only versus lipiodol plus degradable starch microspheres (DSM)).

## Methods

### Patient population

The local ethics committee approved this retrospective study. All patients signed consent prior to the clinical treatment. From January 2007 to April 2015, 99 patients (21 women and 78 men) with HCC underwent repetitive TACE, as predefined by the hospital’s multidisciplinary tumor board. Survival rates were evaluated by reviewing clinical reports until December 2015. By the time of the first chemoembolization session, the mean age was 63.6 years (age range, 34-83 years). Eighty-eight patients had BCLC-Stage-B and in 11 patients, chemoembolization was performed as bridging prior to liver transplantation (BCLC-Stage-A). Hepatitis B was found in 29 (29.3%), hepatitis C in 36 (36.4%), and both in 9 (9.1%) patients. Twenty-six patients had a history of alcohol abuse (26.3%). Three patients (3.0%) had hemochromatosis. 91 patients had liver cirrhosis (Table 1).

**Table 1:** Characteristics of patients with HCC

No. of patients	99
Patients age (years)	63.6 (range: 34-83 years)
Male no. (%)	78 (78.8%)
Female no. (%)	21 (21.2%)
Presence of liver cirrhosis (%)	91 (91.9%)
Initial liver disease:	
Hepatitis B	29 (29.3%)
Hepatitis C	36 (36.4%)
Hepatitis B and C	9 (9.1%)
Hemochromatosis	3 (3.0%)
Alcohol abusus	26 (26.3%)
BCLC-Stage:	
BCLC-Stage A	11 (11.1%)
BCLC-Stage B	88 (91.9%)
Child-Pugh class:	
A	27 (27.3%)
B	72 (72.7%)
Vascular tumor invasion	0%
Confirmation of diagnosis with liver	
Biopsie	99 (100%)
Localisation in liver:	
Right lobar	45% (45.5%)
Left lobe	5 (5.1%)
Bilobar	49 (49.4%)
Number of tumor lesions:	
Single	11 (11.1%)
2	28 (28.3%)
3	8 (8.1%)
4	4 (4%)
Multifocal	48 (48.5%)
Tumor response:	
Partial response	25 (25.3%)
Stable disease	54 (54.5%)
Progressive disease	20 (20.2%)

Fourteen patients (14.1%) were treated because of a recurrence or as advanced therapy of HCC after liver resection, and 4 patients (4.0%) after prior radiofrequency ablation. This population was taken out as a subgroup for analyzing survival time and radiologic response in comparison to the population which received TACE as a first line treatment.

In total, 667 chemoembolization treatments were performed in 4-week intervals with a mean of 6.7 treatments per patient (range: 2-19 treatments per patient). Patients with less than two treatments were not included for evaluation. All patients received 10 mL of the chemotherapeutic agent mitomycin per treatment with slight varieties depending on laboratory techniques. Subjects included between 2007 and 2011 were treated with TACE and lipiodol-only, while patients included between 2012 and 2015 had a combination of lipiodol and DSM. This is caused in a strategy-change in our hospital, regarding a better experience and outcome for the patients.

Patients who met the exclusion criteria prior to the treatment were not included for evaluation. During this retrospective study, no patient was excluded.

### Inclusion criteria

The treatment decision was made by our multidisciplinary tumor board, involving abdominal surgeons, interventional radiologists, and oncologists. In all patients, the presence of HCC was histologically proven by liver biopsy.

In cases that were eligible for liver transplantation (BCLC Stage A), TACE was performed as bridging treatment.

In all patients, we calculated the total volume of all hepatic lesions per patient in addition to the depending liver volume in order to estimate the hepatic tumor load. Only those patients with < 70% hepatic tumor-involvement were treated. To be eligible for TACE, patients had to fulfil certain laboratory and clinical criteria including adequate hematic, hepatic and renal functions in addition to an ECOG performance score of 0 or 1.

Study patients should have had at least 2 treatments of chemoembolization, performed with a 4-week-interval in-between.

### Exclusion criteria

We excluded patients with a hepatic tumor load greater than 70% in order to avoid impairment of the remaining liver function and liver failure. Further, we excluded patients with total thrombosis of the portal vein, patients with extrahepatic metastases and those with renal (creatinine level > 2 mg/dl in serum), hepatic, respiratory, or cardiovascular impairment.

Inadequate performance status as indicated by an ECOG > 1, nutritional impairment, high serum total bilirubin level (> 3 mg/dL), and poor hepatic synthesis (albumin level < 2.0 mg/dL in serum) were further exclusion criteria.

### TACE- therapy

After introduction of a catheter using the Seldinger technique through the femoral artery, an angiography of the abdominal vessels was performed. For angiography a 5F Pig-Tail catheter (Boston Scientific) was used, affiliated by an exchanged over the guide wire with a 5F Side-Winder catheter (Terumo, Tokyo, Japan), used for selective catheterization and angiographic visualization of the superior mesenteric artery and the celiac trunk. After selective catheterization an exploratory overview of the aorta and angiography of the celiac trunk as well as indirect portography were performed. Therefore, contrast media was injected after catheterization to simulate the physiological blood flow. Depending on the size, location, and arterial supply of the tumor, the tip of the catheter was advanced further into tumor-supplying arteries. After positioning the catheter chemotherapeutic agent was administered intra-arterially. The tumor vessels were occluded with embolization agents. After embolization, devascularization was confirmed with additional angiography of the hepatic artery.

In case of involvement of both hepatic lobes, the lobe with the higher tumor burden was treated first. The other lobe was treated in another session of chemoembolization, to keep the risk of liver failure as low as possible.

The chemotherapeutic agent used was mitomycin (Medac®, Hamburg, Germany) alone [10] with a maximum of 8 mg/m^2^ body surface. Mitomycin was applicated with either lipiodol (Guerbet®, Sulzbach, Germany) only, or lipiodol in combination with DSM (EmboCept®S, PharmaCept GmbH, Berlin, Germany) (200-450 mg pro session) in sandwich technique. After finalizing the treatment, a compression bandage or a percutaneous closure device (Angio-Seal™, St. Jude Medical, Saint Paul, USA) was attached on the side of puncture.

For embolization, agents were injected under fluoroscopic guidance until a stasis of bloodflow was achieved.

Patients were transferred to an internal medicine ward for clinical observation subsequent to the treatment and were discharged on the procedure day when no complications were encountered.

Treatment sessions were repeated until the end point of treatment was reached, defined as a state of stable disease situation in two successive sessions. A session covers 3 consecutive TACE treatments, performed with 4 week intervals in-between the treatments. The other endpoint was the case of progressive disease.

In case of PD, patients were reevaluated by our multidisciplinary tumor board for alternative treatment options.

In case of de novo lesions or disease progression during follow-up despite initial tumor stabilization, patients were retreated by using the treatment plan.

### MRI follow-up

MRI follow-up was performed to evaluate the tumor response. For the purpose of planning the intervention, unenhanced and contrast-enhanced MR imaging with the administration of 0.1 mmol/kg body weight of gadoter acid (Dotarem®, Guerbet GmbH, Sulzbach, Germany) or gadobutrol (Gadovist®, Bayer Vital GmbH, leverkusen, Germany) was performed in all patients.

A 1.5-T MRI-system (Magnetom Espree; Magnetom Avanto-fit; Siemens, Erlangen, Germany) was used. Enhanced MR imaging was performed before and 4 weeks after each session. Unenhanced MR imaging was performed prior to each TACE treatment. A monthly follow-up after the endpoint of angiographic treatment was done for a period of 3 months by MRI. After that, this regimen was changed to an interval of 3 months for the entire life of the patients. None of the included patients was lost to follow-up.

Four to six hours after embolization, retention of iodized oil in the HCC lesions was confirmed by unenhanced computed tomography (CT). All CT scans were performed using spiral technique (Somatom Definition AS 128, Siemens, Erlangen, Germany).

### Quantitative and statistical evaluation

Datasets of all patients were evaluated retrospectively. Each clinical data was obtained either by contacting the patients themselves or by contacting their treating physicians. In addition, we reviewed patients’ medical records. Event occurrences were reported.

All MRI and CT readings were performed in consensus by two radiologists, each with more than 5 and 12 years of experience in abdominal imaging. The local tumor response was assessed by MRI, using Response Evaluation Criteria in Solid Tumors 1.1 (RECIST 1.1.). Statistical analysis was performed using BiAs 10.12 software.

Survival times, starting at point of first chemoembolization, were calculated to obtain median and mean survival times by using the Kaplan-Meier method and compared with the log-rank test. Survival rates were calculated in terms of 1-, 2- and 3-year survival. Subgroup analysis and differences in survival between groups were assessed by log-rank test. *P* ≤ 0.05 was considered significant.

## Results

### Local results

Location of the tumor was in 45.45% (45/99) in the right liver lobe, and in 5.1% (5/99) in the left lobe. In 49.45% (49/99), both liver lobes were affected. The number of liver lesions were as follows: 48.5% of patients (48/99) had multiple lesions (≥ 5), 4.0% (4/99) had four lesions, and 8.1% (8/99) had three lesions. In 28.3% of patients (28/99), two lesions were detected and 11.1%11 (11/99) of patients had a singular lesion.

**Fig. 1:**
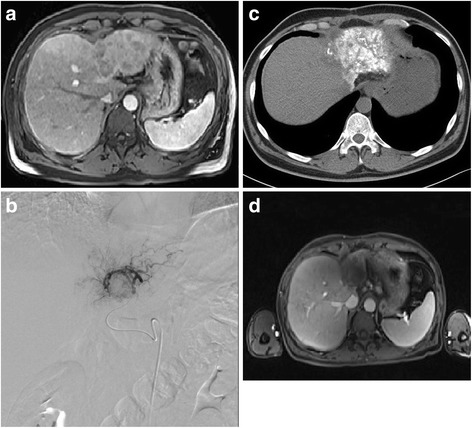
Partial response achieved after six sessions of TACE in a 66-year-old patient with HCC using mitomycin, lipiodol and DSM. **a**. Pre-treatment transverse contrast-enhanced T1-weighted MR image shows an intrahepatic liver lesion affecting both liver lobes. **b**. Prior to TACE procedure, angiographic image shows the presence of hypervascularity of liver tumor. **c**. CT after 3. TACE. Documentation of Lipiodol-deposition in the region of the HCC. **d**. Post-treatment transverse contrast-enhanced T1-weighted MR image after 6 sessions of TACE shows partial response of the intrahepatic lesion

The post-interventional evaluation was based on the RECIST 1.1 und all patients were revealed using this criteria: partial response (PR) (Fig. 1) in 25.3% (25/99), stable disease (SD) in 54.5% (54/99), and progressive disease (PD) in 20.2% (20/99) of all patients.

In the lipiodol only group (51.5%; 51/99), we observed partial response in 21.6% (11/51), stable disease in 62.7% (32/51), and progressive disease in 15.7% (8/51). In the lipiodol-DSM group (48.5%; 48/99), partial response was encountered in 29.2% (14/48), 45.8% (22/48) had stable disease, and progressive disease was observed in 25.0% (12/48) of patients. There was no statistically significant difference between the two treatment protocols (*p* = 0.211) (Table 2).

**Table 2:** Statistical data of study’s patients

	Lipiodol	Lipiodol+EmboCept®S	*p*-value
Number of patients	51	48	0.584
➢ female	13	8	
➢ male	38	40	
Age of patients (years)	63.8	63.4	0.972
Number of TACE	283	384	0.421
Presence of liver cirrhosis (%)	38	53	
Initial liver disease:			
Hepatitis B	10	19	
Hepatitis C	23	13	
Hepatitis B and C	6	3	0.643
Hemochromatosis	1	2	
Alcohol abusus	13	13	
BCLC-Stage:			
BCLC-Stage A	3	5	0.194
BCLC-Stage B	35	56	
Child-Pugh class:			
A	9	18	0.206
B	36	36	
Localisation in liver:			
Right lobar	22	23	
Left lobe	3	2	0.310
Bilobar	19	30	
Number of tumor lesions:			
Single	8	3	
2	28	0	
3	8	0	0.252
4	4	0	
Multifocal	0	48	
Tumor response:			
Partial response	11	14	0.211
Stable disease	32	22	
Progressive disease	8	12	

### Survival analysis

**Fig. 2:**
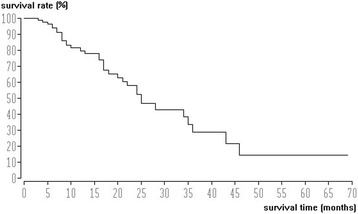
Survival data (Kaplan-Meier method) of patients with HCC (*n* = 99). Median survival time was 28 months from the start of chemoembolization therapy

Median and mean survival times were 28 and 36.4 months, respectively. Survival rate after the endpoint of TACE was 85% at 1 year, 60% at 2 years, and 40% at 3 years per Kaplan Meier evaluation (Fig. 2).

**Fig. 3:**
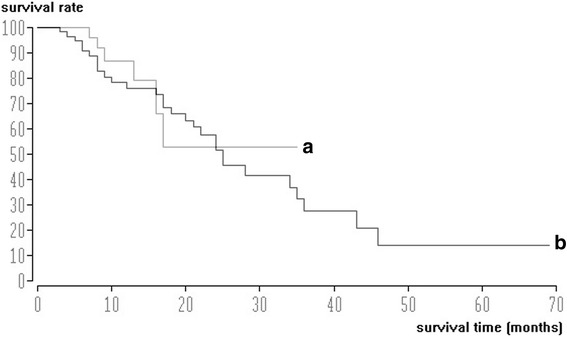
Kaplan-Meier survival curves. **a**. Survival data of all patients with HCC after TACE with lipiodol only (*n* = 51). Median survival time was 25 months. **b**. Survival data of patients with HCC after TACE with lipiodol and DSM (*n* = 48). Median survival time was 28 months

For patients with chemoembolization with lipiodol only, the median and mean survival times were 25 and 28.4 months, respectively (Fig. 3a). Using the combination of lipiodol and DSM for chemoembolization, the median and mean survival times were 28 and 35.7 months (Fig. 3b), respectively. Statistical analysis showed no differences among the groups using the log-rank test (*p* = 0.845).

Median and mean survival for patients with stable disease (SD) were 28 months vs. 27.6 months (lipiodol only vs. lipiodol and DSM), for patients with progressive disease (PD) were 25 months vs. 34.4 months. In case of partial response (PR), 43 months vs. 46.4 months were reported. Analysis showed no statistical differences between the three RECIST-groups using the log-rank test (*p* = 0.146).

Eightteen (18.1%) patients had a recurrence or intensified further therapy of HCC after liver resection or radiofrequency ablation. Analysis of the results from this pretreated subgroup compared with the results of the non-pretreated group showed no statistical significance regarding survival time (*p* = 0.67) and radiological response (*p* = 0.59) and furthermore, no impact on the results.

After the endpoint of TACE, 8.1% of patients (8/99) underwent a liver transplantation, 3.0% (3/99) received liver resection, and 21.2% (21/99) were further treated with ablation (RF or MWA).

Overall, the majority of patients tolerated chemoembolization well and all patients were discharged from the hospital on the day of treatment. However, a small group of patients (*n* = 15) had symptoms of abdominal pain, nausea, and vomiting for 2 to 7 days (“postembolization syndrome”). No major complications or allergic reactions were reported in our patient group.

## Discussion

TACE is one of the most common angiographic option for the treatment of HCC. The applied drugs and embolization agents differ among sites and physicians due to lack of knowledge. A commonly used embolization agent in TACE for the treatment of HCC is lipiodol. Lipiodol is a poppy-seed oil, which is used in interventional radiology as a radio-opacifying contrast agent [10–12]. Due to its chemical characteristics, lipiodol is used in lymphangiography, transarterial embolization (TAE), and chemoembolization (TACE). Furthermore, it is useful in monitoring tumor changes after treatment with TAE or TACE, based on its iodine component allowing the visualization of the tumor and the treated area by CT [12–16]. When injected in the hepatic artery after selective or superselective catheterization, lipiodol remains in tumor nodules for several weeks to over a year, due to a siphoning effect from hypervascularization and neovascularization of tumor vessels and the absence of Kupfer-cells inside the tumor. Lipiodol distributes in the tumor artery branches and the peritumor portal venules, thus allowing transient dual embolization [16, 17].

Degradable starch microspheres (DSM) can also be used as a chemoembolization agent. After injection, in a similar technique as lipiodol, DSM provide transient occlusion of small arteries. Further, DSM may improve therapeutic effect of applied anticancer drugs [18]. The duration of DSM in the tumor vessels is limited to 80 min [19]. DSM is administered until stasis of arterial flow or reflux [20].

Several treatment studies of liver tumors indicated that TACE with a combination of lipiodol and DSM as occlusion agents improve the therapeutic effect of chemotherapeutic agents compared to lipiodol only or treatment using chemotherapeutic agents alone without embolization agents [21]. However, a few studies have evaluated TACE using DSM as a mono-agent in HCC patients [22, 23].

This current single-center study was performed to determine the response and survival rates for patients who underwent TACE of HCC and to compare the effect of two regimens of embolization, namely lipiodol only or in combination with degradable starch microspheres.

Our study shows that TACE with lipiodol and degradable starch microspheres does not result in a significant better local tumor control. However, in patients with multiple liver lesions, a trend towards using the combination for TACE instead of using lipiodol only could be demonstrated.

Considering survival rates, a positive trend, but not a significant better outcome could be shown for using lipiodol and degradable starch microspheres compared to lipiodol-only.

Yamasaki et al. [19] treated 45 patients with HCC using transarterial infusion therapy (TAI) either with lipiodol (*n* = 15), DSM (n = 15), or the combination of lipiodol and DSM (n = 15). The TAI utilized the combination of lipiodol and DSM was superior to TAI using lipiodol or DSM only because of demonstrating improvements in therapeutic effects and progression-free survival. In our study, the documented response rates were 40% in the lipiodol group, 53.4% in the DSM group, and 80% in the lipiodol and DSM group. No significant differences between the three groups were seen regarding the survival rates.

We found a response rate of 21.6% in the lipiodol only group, and 29.2% in the lipiodol plus DSM group. Treatment response in the lipiodol plus DSM group was superior compared to the lipiodol only group, but this result showed no statistical significance, as it was shown in the study of Yamasaki et al..

Some of the discrepancy may be attributed to the performed technique of TACE, such as treatment with cisplatin in the study of Yamasaki et al. compared to the use of mitomycin in our study. Furthermore, all patients of Yamasaki et al. had up to 6 treatment sessions. In our study, 667 chemoembolization procedures were performed (mean, 6.7 treatments per patient; range, 2-19 sessions).

It has been stated that the assessment of tumor response after one TACE session is very critical and questionable, and that at least two TACE sessions should be performed in the same targeted lesions before further treatment is abandoned [24, 25].

In the study of Kirchhoff et al. [24], 47 patients received TACE with DSM and lipiodol. Doxorubicin and cisplatin were used as chemotherapeutic agents. Median survival rate was 26 months and 1-year survival rate was 75%, which is comparable to our results.

Achenbach et al. [25] performed TACE in 22 patients using mitomycin and lipiodol. Median survival of patients in this study was 14 months with 1- and 2- year survival rates of 69% and 29%, respectively. The investigators reported shorter survival times and lower survival rates compared to our study, when using mitomycin as chemotherapeutic agent, similar to our study, but lipiodol only for embolization.

Dumortier et al. [26] evaluated 89 patients with unresectable HCC, treated with chemoembolization. Treatment included up to six cycles of hepatic intra-arterial application of lipiodol with doxorubicine and gelatine sponge. The median survival was 13 months with a 4-year survival rate of 13.6%. The discrepancies may be attributed to the used chemotherapeutic agent (doxorubicin versus mitomycin), different occlusion agents (lipiodol plus gelatin sponge versus lipiodol or lipiodol plus DSM), or the amount of TACE sessions.

Notably in our and priorly published studies, radiological response to the therapy was not associated with improved survival. Superior response suggests, that TACE using DSM and lipiodol could be a suitable palliative treatment in patients with HCC [18, 24, 27].

The current study has several limitations. First, the study design was retrospective. Second, there was an overlap between the palliative indication and the neoadjuvant indication of chemoembolization. Last, more patients should have been recruited and a prospective randomized study would be more accurate to assess treatment safety and efficacy of TACE with lipiodol versus DSM.

## Conclusion

Our data suggest a slight benefit of the use of lipiodol and DSM in comparison of using lipiodol only for chemoembolization of HCC in terms of local tumor control and survival data, this trend did not reach the level of significance.

## Abbreviations

BCLC staging: Barcelona clinic liver cancer staging
CT: Computed tomography
DEB-TACE: Transarterial chemoembolization with drug-eluting-beats
DSM: Degradable starch microspheres
ECOG: Eastern cooperative oncology group
HCC: Hepatocellular carcinoma
MRI: Magnetic resonance imaging
MWA: Microwave ablation
PD: Progressive disease
PR: Partial response
RECIST: Response evaluation criteria in solid tumors
RFA: Radiofrequency ablation
SD: Stable disease
TACE: Transarterial chemoembolization
TAE: Transarterial embolization
TAI: Transarterial infusion therapy
